# A severe presentation of vitamin D-dependent hypocalcemic rickets associated with hypophosphatemia

**DOI:** 10.1590/2175-8239-JBN-2023-0044en

**Published:** 2023-10-06

**Authors:** Gonçalo Vale, Leonor Cardoso, Telma Francisco

**Affiliations:** 1 Centro Hospitalar Barreiro-Montijo, Barreiro, Portugal.; 2 Centro Hospitalar Universitário Cova da Beira, Covilhã, Portugal.; 3 Centro Hospitalar e Universitário Lisboa Central, Lisboa, Portugal.

This is a case report of a seven-year-old girl evacuated from Sao Tome and Principe due to rickets. She started walking at age 3, was unable to walk since age 4, and had recurrent episodes of respiratory distress^1^. At physical examination, she was malnourished, had bell-shaped thorax, deformed and varus limbs, and missing teeth. Blood tests revealed severe hypocalcemia (6.4 mg/dL) and hypophosphatemia (2.5 mg/dL), high alkaline phosphatase (4161 U/L) and parathyroid hormone (509.6 pg/ml), normal range 25-hydroxyvitamin D; 1,25-dihydroxyvitamin D was not performed. Genetic study identified mutations in both CYP27B1^2^ gene copies, leading to alterations in alpha-1-hydroxylase function. A diagnosis of vitamin-D-dependent rickets type 1A was made.

**Figure 1:**
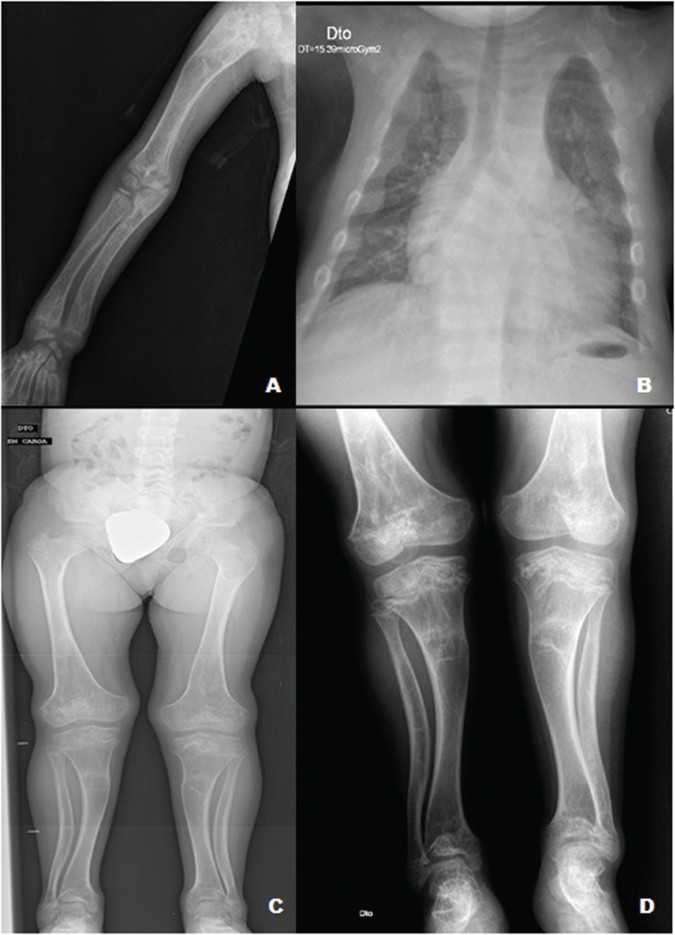
Patient radiographies showing severe bone deformities, pseudo-fractures in upper and lower limbs, and a bell-shaped thorax.
